# Group prenatal care successes, challenges, and frameworks for scaling up: a case study in adopting health care innovations

**DOI:** 10.1186/s43058-024-00556-1

**Published:** 2024-03-04

**Authors:** Claire Masters, Rogie Royce Carandang, Jessica B. Lewis, Ashley Hagaman, Rebecca Metrick, Jeannette R. Ickovics, Shayna D. Cunningham

**Affiliations:** 1 Department of Health Policy and Management, Yale School of Public Health, New Haven, CT 06510 USA; 2 Department of Public Health Sciences, University of Connecticut School of Medicine, Farmington, CT 06030 USA; 3 Department of Internal Medicine, Yale School of Medicine, New Haven, CT 06519 USA; 4 Department of Social and Behavioral Sciences, Yale School of Public Health, New Haven, CT 06510 USA; 5 Center for Methods in Implementation and Prevention Sciences, Yale University, New Haven, CT 06510 USA; 6 Sinai Urban Health Institute, Chicago, IL 60608 USA

## Abstract

**Background:**

Group prenatal care enhances quality of care, improves outcomes, and lowers costs. However, this healthcare innovation is not widely available. Using a case-study approach, our objectives were to (1) examine organizational characteristics that support implementation of *Expect With Me* group prenatal care and (2) identify key factors influencing adoption and sustainability.

**Methods:**

We studied five clinical sites implementing group prenatal care, collecting qualitative data including focus group discussions with clinicians (*n* = 4 focus groups, 41 clinicians), key informant interviews (*n* = 9), and administrative data. We utilized a comparative qualitative case-study approach to characterize clinical sites and explain organizational traits that fostered implementation success. We characterized adopting and non-adopting (unable to sustain group prenatal care) sites in terms of fit for five criteria specified in the Framework for Transformational Change: (1) impetus to transform, (2) leadership commitment to quality, (3) improvement initiatives that engage staff, (4) alignment to achieve organization-wide goals, and (5) integration.

**Results:**

Two sites were classified as adopters and three as non-adopters based on duration, frequency, and consistency of group prenatal care implementation. Adopters had better fit with the five criteria for transformational change. Adopting organizations were more successful implementing group prenatal care due to alignment between organizational goals and resources, dedicated healthcare providers coordinating group care, space for group prenatal care sessions, and strong commitment from organization leadership.

**Conclusions:**

Adopting sites were more likely to integrate group prenatal care when stakeholders achieved alignment across staff on organizational change goals, leadership buy-in, and committed institutional support and dedicated resources to sustain it.

**Trial registration:**

The *Expect With Me* intervention’s design and hypotheses were preregistered: https://clinicaltrials.gov/study/NCT02169024. Date: June 19, 2014.

**Supplementary Information:**

The online version contains supplementary material available at 10.1186/s43058-024-00556-1.

Contributions to the literature

- Profound disparities in perinatal morbidity and mortality persist. Prenatal care has remained largely unchanged for a century, despite promising innovations like group prenatal care.
- We apply the Framework for Transformational Change to assess implementation of group prenatal care, offering a structured approach to characterize adopters and non-adopters using five key criteria.
- Adopting sites were more likely to integrate group prenatal care when stakeholders achieved alignment across staff on organizational change goals, leadership buy-in, and committed institutional support and dedicated resources to sustain it.

## Introduction

Adverse birth outcomes in the United States are higher than all other developed countries, with persistent racial disparities in maternal morbidity and mortality [1, 2]. Prenatal care is critical to achieving optimal health outcomes [3]; however, prenatal care in the US has changed little since its inception [4]. Research has focused on the timing of initiation and number of prenatal care episodes (adequacy), although more visits do not necessarily align with the receipt of recommended guideline-based care [5]. Group prenatal care is a promising alternative to improve perinatal outcomes [6, 7].

Traditional prenatal care in the US involves one-on-one visits between patients and providers, with appointments lasting 10 min on average [7]. In group prenatal care, a credentialed provider (e.g., physician, midwife, nurse practitioner) delivers care to 8–12 pregnant individuals simultaneously during a group visit that may last up to hours. Group prenatal care maintains one-on-one risk screening and physical assessment of individual prenatal care while using the group format to enhance patient education and social support [8]. Compared to standard individual prenatal care, group prenatal care provides substantially more contact with providers (approximately 2 versus 20 h), enables patients to be actively engaged in their healthcare, provides support services, and is integrated to respond to the complex needs of pregnant people and their families. Centering Pregnancy [9], *Expect With Me* [10], Supportive Pregnancy Care [11], and individual practices have devised their own group prenatal practices.

Evidence for CenteringPregnancy and *Expect With Me* specifically suggests that, compared to traditional individual prenatal care, group prenatal care is associated with positive outcomes including decreased rates of preterm birth, increased birth weight in preterm infants, increased breastfeeding initiation and duration [12–15], greater use of postpartum family planning services [16, 17], and fewer emergency room visits in the third trimester of pregnancy, for largely low risk pregnant individuals [18]. Group care patients have better prenatal knowledge, feel better prepared for labor and delivery, and both patients and providers are more satisfied with care [19, 20]. Investments in group prenatal care reduce costs with fewer admissions to Neonatal Intensive Care Units (NICU) [21–23].

Despite potential benefits of broader implementation, access to group prenatal care remains limited. When informed of care options, many pregnant women would likely enroll in group prenatal care if offered [24, 25]. However, implementing and sustaining group prenatal care can be challenging [26, 27]. On the other hand, there are potential cost savings associated with these models of care [21, 22], warranting a better understanding of these implementation efforts. It can be difficult to implement organizational change even when healthcare innovations are evidence-based, and up to two thirds of change efforts fail [28, 29]. Substantial effort is required to transform clinical protocols, clinic workflow, and scheduling as well as for staff training and promotion of group prenatal care [30].

Given the gap between potential benefits of group prenatal care as a healthcare innovation and widespread implementation, the objective of this paper is to evaluate healthcare system characteristics that impact uptake of *Expect With Me* group prenatal care. Several frameworks have been proposed for scaling up healthcare innovations. We apply an adapted version of the Framework for Transformational Change in Healthcare to guide our analyses [31]. This framework specifies the following five criteria must be present to achieve successful organizational change: (1) impetus to transform, (2) leadership commitment to quality, (3) improvement initiatives that actively engage staff in meaningful problem solving, (4) alignment to achieve consistency of organization-wide goals with resource allocation and actions, and (5) integration to bridge traditional intra-organizational boundaries between individual components. We utilized a comparative qualitative case-study approach [32] to characterize clinical sites in terms of their fit to these criteria, and explain organizational traits that fostered implementation success.

## Methods

We used a comparative qualitative case study approach to explore implementation of *Expect With Me* group prenatal care across five clinical sites in Nashville TN, Detroit MI, and McAllen TX as part of a translational research study (2014–2017). Described in detail elsewhere [10, 33], *Expect With Me* is delivered by a prenatal care provider (facilitator) and a co-facilitator, usually a medical assistant or nurse, and consists of ten 2-h sessions that follow clinical guidelines from the American College of Obstetricians and Gynecologists [34]. Unique features of *Expect With Me*, relative to other group prenatal care models, include greater emphasis on content pertaining to nutrition, physical activity, sexual health, stress, and mental health, and a HIPAA-compliant integrated technology platform. The technology platform enables group engagement to extend beyond in-person interactions, encouraging patients to monitor their health behaviors and to connect with other participants and their providers. Patients can track health goals, join group discussions, message and chat with others, obtain resources, and watch educational videos. The IT platform provides a useful tool for providers to monitor attendance, upload and distribute educational materials to patients between visits, document care/content delivered, identify patient needs, and plan targeted care for clients [10]. A scheduling tool is available to clinics to account for provider time, group space and clinic schedules [10].

In preparation to implement *Expect With Me*, organizations undergo a readiness planning and change management process, followed by in-depth training for clinical staff and providers. A roadmap guides clinical sites to understand the system-wide support and logistic requirements for a successful group care practice. Change management and communications templates, webinars, videos, reading lists, and pre-work packages provide a grounding in group prenatal care structure, content, and research basis. An in-person organizational training session follows with all levels of clinic staff to introduce the model of care and review new processes at the clinical site for enrolling and managing patients in a group care practice and to train staff on the accompanying IT platform. A 1- to 2-day facilitator training with providers and staff teaches participants how to conduct a group prenatal visits, including brief individual medical checks, engaging patient in self-care activities, and facilitating group sessions. Participants learn facilitative leadership skills, become familiar with the curriculum, and practice leading activities that engage patients in learning. Sites receive ongoing technical assistance.

### Data sources

Data were obtained from administrative records, the *Expect With Me* technology platform, focus groups, and semi-structured interviews. A triangulated approach to data collection is ideal for studying organizational change, allowing for comprehensive examination of organizational processes [35].

#### Site profiles and implementation data

Information about health center size and staffing, patient volume, birth outcomes, and patient demographics were obtained from administrative records to develop site profiles that reflect the state of each health center at the start of the implementation study. The *Expect With Me* information technology platform captures real-time program implementation data for every prenatal care group including location, facilitators, co-facilitators, session dates, and times that were used to assess frequency and fidelity of group prenatal care delivery at each site.

#### Focus groups and semi-structured interviews

Between June and September 2015, approximately 1 year after initiating delivery of *Expect With Me* group prenatal care, four 1-h focus groups with 41 healthcare providers and administrators (e.g., clinic managers, medical directors, hospital administrators) were conducted by the Yale principal investigator and other trained study staff during site visits. In addition, eight semi-structured interviews (60–90 min each) were conducted with clinicians and staff members implementing group prenatal care. Interviews were completed with participants best situated to observe group care at their site. During the focus groups and interviews, participants were asked about advantages and disadvantages of group prenatal care, challenges to implementation, adaptations at their site, the technological platform, institutional support, technical assistance from the trainers, and sustainability of the model.

#### Analytic approach

Sites were categorized dichotomously as “adopters” and “nonadopters” for the purposes of these analyses according to the extent of group prenatal care implementation. We categorized sites as adopters based on the following criteria: running an average of two new groups per month, beginning a new group at least every 2 months, consistently conducting groups for 12 months or more, and designating a primary facilitator and co-facilitator for each group.

Adopters and non-adopters were then characterized using the Framework for Transformational Change in health care developed by VanDeusen Lukas and colleagues (2007), which specifies the following five criteria must be present to achieve successful organizational change: (1) impetus to transform, (2) leadership commitment to quality, (3) improvement initiatives to engage staff, (4) alignment to achieve organization-wide goals, and (5) integration. The analysis was deductive based on these five domains. Additional organizational factors that may facilitate adoption of group prenatal care were identified inductively using an interpretive description methodology, tailored for use in healthcare research and involves exploring patterns and relationships. The qualitative interviews and focus group discussions were transcribed and coded in DEDOOSE v. 4.2, according to predetermined categories as well as themes that arose inductively. Inductive codes included: benefits to providers [of group prenatal care], buy-in to group prenatal care, and challenges to implementation. These codes allowed for additional characterization of adopters and non-adopters. We ensured qualitative rigor in several ways, including using constant comparison to ensure the credibility of results, paying attention to alternative explanations beyond our deductive framework; ensuring dependability by using researcher memos to document changing contexts and circumstances during study implementation; and considering the research team’s own positionality vis-à-vis data collection and interpretation of results through reflexive practices at team meetings. Coding was led by RM, who is a white female and was a Master’s degree student in public health at the time of the study. Codes were reviewed and discussed with SDC and JRI, both white female doctorally trained faculty members, and any discrepancies were resolved upon consultation between additional co-authors: CM, a master’s degree level trained female staff member employed at a school of public health, JPL and AH, also white female doctorally trained faculty members, and RRC, a Southeast Asian male doctorally trained postdoctoral fellow.

This study was approved by the Institutional Review Boards at the Yale School of Medicine (HIC #1,304,011,772) and all participating hospital/university systems. Staff explained the study to participants, answered questions, and obtained informed consent. Participants received a $20 gift card as compensation for their time. Standards for Reporting Qualitative Research (SRQR) guidelines were followed.

## Results

### Site characteristics

Table 1 provides a description of each site and details about its implementation of *Expect With Me* during the study period. These sites represent a variety of organizational structures and are diverse in terms of geography, patient population, and volume. Although all sites initiated group prenatal care delivery, two had stronger uptake as demonstrated by their ability to initiate new groups more frequently and over a longer timeframe. One adopter was an obstetrics and gynecology clinic at a high-volume urban academic medical center that successfully ran at least one new group each month over 22 months with 23 clinical and administrative staff members facilitating groups. The other was a low-volume urban perinatal research clinic within an academic medical center that initiated new monthly groups across 15 months with16 staff members facilitating groups. Two of the non-adopter sites were smaller satellite hospitals of the main study sites. The other non-adopter site employed a unique “feeder model,” whereby physicians in private practices referred patients at their initial intake visit to receive group prenatal care at the local community hospital.

**Table 1:** Description of sites and implementation (2013)

Site	Urban/rural	Percent publicly insured patients	Staffing	Implementation timeframe
**Adopters**				
A	Urban academic medical center	40% Medicaid	• 23 total staff facilitating • 13 providers facilitated on average 4.5 groups each • 10 staff co-facilitated on average 5.2 groups each	• 53 groups over 22 consecutive months • At least one group starting per month
B	Urban research clinic within academic medical center	90% Medicaid	• 16 total staff facilitating • 11 providers facilitated on average 4.8 groups each • 5 staff co-facilitated on average 10.6 groups each	• 53 groups over 15 consecutive months • At least one group starting per month, except during two months in the first year
**Nonadopters**				
C	Urban academic medical center, “satellite” site for *Expect With Me*	95% Medicaid	• 33.3% of groups did not have a primary co-facilitator • 10 staff were involved in facilitating or co-facilitating groups • 5 providers facilitated on average 7.2 groups each, and 5 staff co-facilitated on average 4.8 groups each	• 36 groups over 15 months. Gaps of 1–3 months with no new groups
D	Rural community hospital, “satellite site” for *Expect With Me*	Data unavailable	• 7.8% of groups did not have a primary co-facilitator • 7 staff were involved in facilitating or co-facilitating groups • 5 providers facilitated on average 2.6 groups each, and 2 staff facilitated on average 6 group each	• Ran 13 groups over 17 months. Gaps of 1–2 months with no new groups
E	Prenatal care delivered at private offices, “feeder model” for *Expect With Me*	85% Medicaid	• 90% of groups did not have a primary co-facilitator • 2 staff were involved in facilitating or co-facilitating groups • 1 provider facilitated all 10 groups, and 1 staff member co-facilitated one group	• Ran 10 groups over 8 months

### Framework of Transformational Change—model fit

Adopters demonstrated better fit with all five criteria articulated by the Framework of Transformational Change, with some criteria holding greater importance to meeting implementational goals. Results are organized by the five key elements of this organizational change model, comparing and contrasting adopting and non-adopting sites (see Table 2 for synthesis).

1. *Impetus to transform care.* The impetus to transform prenatal care delivery at adopting sites was based on the belief that providing group prenatal care would draw new patients and improve patient education, clinic efficiency, provider satisfaction, and cost-effectiveness. In contrast, participants from non-adopter sites reported that while they were motivated to deliver group prenatal care as a means of improving patient experience and outcomes, they did not expect increases in efficiency or cost-effectiveness, suggesting they may have had a narrower conception of benefits. At one adopter site, *Expect With Me* was initially championed by hospital administrators, whereas at the other it was by clinicians. However, in both cases, the individuals promoting this change were able to effectively communicate their vision to other stakeholders within their respective organizations to gain buy-in. These conversations focused on evidence for the benefits of group prenatal care and resulted in multilevel consensus that implementing *Expect With Me* represented,

**Table 2:** Key elements of organizational transformation to deliver high-quality group prenatal care

Key themes	Description	Adopters	Non-adopters
Impetus to transform	Motivation can arise either externally, influenced by external pressures, or internally, driven by a myriad of factors within the organization	**Multiple benefits** *“Providing group prenatal care would draw new patients and improve patient education, clinic efficiency, provider satisfaction, and cost-effectiveness of care.”* **Cultivating advocates** *“Group prenatal care was initially championed by hospital administrators or clinicians.”* **Effective communication** *“We communicated our vision to other stakeholders within our respective organizations to gain buy-in.”*	**Did not anticipate efficiency improvements** *“While we are motivated to deliver group prenatal care as a means of improving patient experience and outcomes, we did not expect increases in efficiency or cost-effectiveness of care.”* **Top-down approach lacks complete buy-in from health care providers** *“While administrators were said to be on board, the message did not reach those responsible for making referrals.”*
Leadership commitment to quality	Leadership commitment begins at the top of the organization but includes all levels	**Leadership and support by senior clinicians** *“We had senior clinicians overseeing group prenatal care implementation who could draw on their own experiences facilitating group sessions to provide support to other health care providers.”* **Hospital leadership** *“Adequate space, including a meeting area spacious enough for all participants, was provided for the group sessions, in contrast to the confines of an examination room.”*	**Lack of leadership buy-in** *“How can you implement a new model of care when you cannot even implement regular care?*
Improvement initiatives that engage staff	Improvement initiatives mobilized diverse staff at all levels to solve a pressing and meaningful problem collaboratively	**Implementation of an “opt-out” policy for group prenatal care** *"The head of our obstetrics department introduced an 'opt-out policy' for patient scheduling, wherein eligible individuals were primarily presented with the option to enroll in group prenatal care unless they chose otherwise."* **Collaborative efforts for optimizing group prenatal care** *"We collaborated on various initiatives to enhance group prenatal care, including evaluating and shifting protocols aimed at improving the recruitment and retention of patients."*	**Staff disengagement toward new initiatives** *"How can we earnestly drive change when resources are limited?"*
Alignment to achieve organization-wide goals	Consistency of plans, processes, information, resource decisions, and analysis to support key organization-wide goals	**Resource allocation and actions** *“We have dedicated resources to enhance health outcomes, including personnel, time, supplies, and space. However, there needs to be more consistency in aligning goals and expectations for implementing this supplementary care model.”* *“…It is problematic to introduce, try to sell [group prenatal care] when you are using the traditional model.”* *"To fully integrate and sustain group prenatal care, aligning financial incentives is essential."*	**Inability to adjust processes and redistribute resources** *“We did not have a designated space and that was a huge, huge obstacle.”*
Integration	Integration involves aligning structures and processes to facilitate the widespread adoption of improved clinical practices across all organizational levels	**Systematically incorporated into the services** *"We provide information about group prenatal care to every new patient, including those identified as high-risk."* **Institutional barriers** *“The new program exists in addition to the clinic’s regular services.”* *“… It will never be a default as long as you designate it as something alternative.”* **Challenges in sustainability** *“We are unable to sustain the model after a significant leadership change.”*	**Institutional barriers** *"Innovations often require additional financial and logistical resources."* **Misconception** *"I believe this innovation is best suited for specific patient profiles, particularly those with high social risk factors."* **Resistance to collaborative patient care** *“At the end of the day, the physicians just did not want to give up control of their patients.”*

“A great opportunity to be able to see if this [group prenatal care] is going to make a difference in the outcomes of women who really need this type of intervention.” (Health care provider, adopting site, focus group).

In contrast, the impetus to transform at the non-adopting sites was largely driven by a top-down approach from hospital administrators without complete buy-in from health care providers. According to one participant,

“All of the administrators had supposedly bought into it, but none of that was communicated down to the people who were actually sending the referrals.” (Administrator, non-adopting site, focus group).

Effective, clear communication channels were a key mechanism in fostering the impetus to transform care from site leadership to all levels of staff.

(2) *Leadership commitment.* Hospital leadership ensured adequate meeting space was provided, that is, a room large enough to hold all group participants comfortably. A health care provider at an adopting site said,

“[Hospital] administrators enhanced facilities for delivery of group prenatal care. [They] went above and beyond to make it [group prenatal care] really successful and really nice for all patients.” (Health care provider, adopting site, focus group).

Both adopting sites had senior clinicians overseeing group prenatal care implementation. They provided support to other health care providers based on their own experience facilitating group care. At one site, the chair of obstetrics implemented an “opt out” policy for scheduling patients, whereby all eligible patients were offered the primary option of enrolling in group prenatal care unless they declined. According to an administrator at an adopting site:

Once leadership committed to implementing group prenatal care, “everyone was doing what they’re supposed to be doing to make sure it happens.” (Administrator, adopting site, semi-structured interview).

(3) *Improvement initiatives that actively engage staff*. Commitment from senior clinical leadership in designating group prenatal care as the primary option built confidence and trust. At adopting sites, health care providers and clinic staff also collaborated in continuous quality improvement efforts to enhance group prenatal care delivery. This included on-going evaluating and willingness to change clinical protocols as needed to improve patient recruitment and retention. In contrast, non-adopting clinical sites faced resistance to any change, citing administrative burden and resource constraints.

“How can you implement a new model of care when you can’t even implement regular care.” (Administrator, non-adopting site, focus group).

At non-adopting sites, lack of leadership buy-in contributed to staff disengagement toward new clinical initiatives such as group prenatal care. They just wanted to “get their regular jobs done.”

(4) *Alignment to achieve consistency of organization-wide goals with resource allocation and actions*. Adopters reported alignment between organizational goals (e.g., provide good prenatal care, improve birth outcomes), allocation of resources (such as personnel, time, supplies, space), and successful implementation of *Expect With Me*. Adopters had strong institutional support, including someone responsible for coordinating all aspects of group prenatal care and dedicated space for group visits. In contrast, non-adopting sites did not have the capacity to shift organizational processes and reallocate resources to support even the most basic changes required to implement group prenatal care. For example, one participant reported,

“We didn’t have a designated space and that was a huge, huge obstacle.” (Health care provider, non-adopting site, focus group).

Even among adopters, time constraints could be challenging.

“Group prenatal care requires focused time to make sure all the materials are available and time to actually facilitate the program as it was meant to be done.” (Health care provider, adopting site, focus group).

Participants noted that sustaining group prenatal care requires willingness to redefine expected productivity measures for health care providers. One described how providers would return from facilitating a group session and “feel behind clinically,” because productivity metrics were based on a traditional individual care structure. This expectation can be a disincentive to facilitate groups that are small or where attendance is poor. To enhance patient engagement, participants emphasized that intake appointments for new patients be scheduled with providers who are facilitating the next prenatal care group, so that they do not have to be transferred to a new provider to be placed in a prenatal care group. One participant explained,

“I try to build rapport with [new patients]. What I find is that after I build rapport with them in individual setting, they say well, what the hell, I’ll just see you, ‘cause I feel comfortable asking you questions…It’s problematic to introduce, try to sell [group care], when you’re using the traditional model to bring the person into the system.” (Health care provider, adopting site, focus group).

Participants articulated the importance of aligning organizational change goals with resource allocation, including modifying clinic practices to build trust and rapport between patients and providers. Thus, facilitating implementation of alternative care models.

Participants noted that fully integrating and sustaining group prenatal care will require alignment of financial incentives so that monetary savings from improved perinatal and neonatal outcomes attributable to group prenatal care can be re-invested in organizational change efforts. Current payment models that do not tie reimbursements to outcomes pose a barrier to the uptake and maintenance of group prenatal care and other healthcare innovations.

(5) *Integration of Expect With Me into regular clinic operations*. Integration of a new (i.e., additional) prenatal care model proved a challenge for both adopters and non-adopters. Informants indicated that this may be because additional financial and operational resources are commonly devoted to innovations that are considered ‘special programs’. While these supports are assets to initial adoption, they can be barriers to integration, since the program exists *in addition to* regular services. One participant from an adopter site noted that when group prenatal care is funded through a grant, it is seen as ancillary. There may be an ‘adjunctive effect’ whereby administrators view group prenatal care as a temporary offering, rather than deeply embedded.

“If you are trying to introduce a model of care that you believe should be the default, it will never be the default as long as you designate it as something alternative.” (Health care provider, adopting site, focus group).

One example of the difference in level of integration of group prenatal care between adopting and non-adopting sites was how frequently patients were invited to participate in group prenatal care. At adopting sites, providers made a concerted effort to share information about group prenatal care with every new patient, including high-risk patients. Group prenatal care was systematically incorporated into the services they offered. At one non-adopting site, providers were more likely to think of group prenatal care as only suitable for certain types of patients (e.g., low medical risk, nulliparous, high social risk). At another, clinicians were reticent to share responsibility for patients. In the words of one participating health care provider from a non-adopting site,

“At the end of the day, the physicians just didn’t want to give up control of their patients.” (Health care provider, non-adopting site, semi-structured interview).

Even in highly active adopter sites, group prenatal care was not sustained beyond the funded study period. Transitioning from research-supported implementation to ongoing clinical practice requires strong administrative support and sustained funding. External incentives for initiatives, such as enhanced reimbursements for group care visits, value-based care structures that reward positive patient outcomes, or policy changes that incentivize outcome or equity metrics may be required to ensure stability and sustainability of innovative, evidence-based clinical programs.

Influential leaders at the two “adopter” clinical sites, who served as champions for the model, left these practices near the end of the study period. One site accepted funding to test a competing model of group care. Another clinical site was unable to sustain group care after a leadership change. Key champions often lead the charge for organizational transformation; therefore, loss of these champions can stall transformation. Integration into regular clinical operations may require sustained external incentives, such as enhanced reimbursement for group prenatal care, value-based care models, accreditation tied to outcome or equity metrics, or other policy changes that focus on patient outcomes or disparities.

## Discussion

Successful implementation of group prenatal care requires many organization-level elements to be in place. While transformational change in a health care organization may require all five characteristics to be addressed—impetus to transform, leadership commitment to quality, improvement initiatives that engage staff, alignment to achieve organization-wide goals, and integration—they may not all need to be present equally. Adopters’ case studies illustrate that implementing an innovative health care delivery model is possible and does not require uniformity in organizational structure, patient population, or culture.

Additionally, the five elements are not independent: they are “interactive and iterative” [31]. Each organizational element can foster the others, or a lack of one can be a barrier to achieving a high level of another. Prior research has identified organizational barriers and facilitators to implementing group prenatal care, including structural features (space, resources, staffing, patient volume, community) and attitudinal features (motivation, leadership, buy-in, anticipating change, climate, communication) [27]. This study corroborates and extends those findings by characterizing sites that implemented group prenatal care according to a framework for change in health care organizations.

Despite its promising potential, group prenatal care did not achieve long-term sustainability beyond the study period. Ultimately, without a system-wide commitment to a particular innovation, efforts can be derailed by changes in leadership and personnel (e.g., departure of champions of an innovation) or changes in incentives and funding (e.g., seeking new funding opportunities that result in testing different clinical practices).

Group prenatal care continue to gain traction as an alternative to traditional care. As of January 2024, of the most recognized group prenatal care models in the United States, CenteringPregnancy reported 350 sites in 44 states [36], and Supportive Pregnancy Care had 32 sites in 15 states [37]. An expanding group prenatal care model within the United Kingdom’s National Health Service, ‘Pregnancy Circles,’ is currently being evaluated in an ongoing randomized controlled trial to assess infant outcomes and cost-effectiveness [38]. Few alternative models of prenatal care have achieved successful national scale up, with the exception of New Zealand’s midwifery-led continuity of care model [39].

Scaling up group care may be possible through grants and other special programs, yet sustaining group prenatal care requires broader systemic change. Fee-for-service payment models incentivize volume of care rather than quality or value [40]. Savings that result from innovations primarily accrue to payers (private and public insurers) rather than to health care providers. Therefore, providers have few financial incentives to adopt innovations that result in improved quality and outcomes [41].

Despite the clear importance of dedicated resources and adequate reimbursement models for the enduring success of group prenatal care, there are currently no provisions for federal funding for alternate models of prenatal care. Value-based payment for prenatal care is isolated to a few state Medicaid programs in Ohio, Tennessee, and Arkansas [42]. In South Carolina, a quality improvement partnership between private and public stakeholders led to decreases in preterm birth, in part through enhanced provider reimbursement for group prenatal care [43]. Of the emerging alternative payment models [40], shared savings models that impact provider reimbursement could effectively offset costs of implementing group prenatal care through reinvestment at the source of care.

Healthcare organizations that are responsive to quality improvement initiatives and achieve the alignment of all stakeholders around these goals are most likely to integrate new models of prenatal care into their ongoing practice. Institutional buy-in and leadership, particularly from health care providers, proved essential in initiating change and fueling sites’ transition along a path toward integration into regular clinic operations. However, providers also were hesitant to give up control of patients in group prenatal care, as it challenged the traditional one-on-one model of care as well the notions of productivity entrenched in those models. Despite these barriers, the role of group prenatal care as a tool to improve birth outcomes and reduce the costs of care at a health systems level, is deserving of further exploration.

## Limitations

We focus on group prenatal care across five sites in only three regions of the US, limiting generalizability to other regions, populations, and health care settings. A comparative case study approach is ideally suited to greater than five sites to achieve thematic saturation. Our study utilizes small focus groups and semi-structured interviews with key participants which could introduce biases in sampling of participants and experiences captured. While we report overall staffing engaged in implementation efforts, the exact breakdown of provider mix facilitating group sessions was unavailable to us However, our qualitative design allows for deep insights into site characteristics and organizational factors that facilitated or hindered adoption of group prenatal care, and benefits from the inclusion of multiple stakeholders’ perspectives, including health care providers, administrators, and other actors involved in the implementation process. Moreover, our study utilized a recognized framework for assessing the implementation of new health care interventions, enhancing the validity of our findings.

## Conclusions

Traditional prenatal care models in the United States have existed without major changes for the past century, despite sub-optimal outcomes and persistent disparities. Although there is immense potential for alternative models such as group prenatal care to improve public health outcomes, many organization-level and systemic barriers hinder long term sustainability. Implementation factors that could drive long term sustainability include secure sustainable funding, clinical champions and leaders who can drive change, and robust organizational support. Additionally, ongoing research, evaluation, and collaboration between healthcare providers, policymakers, and stakeholders is essential to refine and expand promising alternative models of prenatal care.

### Supplementary Information

**Supplementary Material 1.**

**Supplementary Material 2.**

## Data Availability

The data supporting the conclusions of this article are available upon request.

## Abbreviations

ACOG: American College of Obstetricians and Gynecologists
AAP: American Academy of Pediatrics
FFS: Fee-for-service
NICU: Neonatal intensive care unit
SRQR: Standards for Reporting Qualitative Research
