# Supplementary material for: Group prenatal care successes, challenges, and frameworks for scaling up: a case study in adopting health care innovations
Source: Implement Sci Commun. 2024 Mar 4;5:20. doi: 10.1186/s43058-024-00556-1 (PMC10913654; doi:10.1186/s43058-024-00556-1)
Supplement: Supplementary file 1 — Supplementary Material 1. [file 43058_2024_556_MOESM1_ESM.docx]

**Clinic Staff Indepth Interview Guide**

1. Tell me about your experience with Expect With Me.

- What do you think are advantages of group prenatal care?
  - Probe about perceived benefits for patients, providers, institutions
- Describe challenges that you or your organization has faced implementing Expect With Me and how you overcame them or what you tried that didn’t work.
  - Probe about:
    - recruitment/retention
    - staff turnover
    - organizational and logistic (e.g., space, scheduling) issues
- Describe factors that positively or negatively influenced implementation at your site.
  - Probe about
    - stakeholder involvement
    - change management process
    - administrative and managerial support
    - champions and respected colleagues
    - innovation attributes
    - Probe about how other providers, support staff and clinic leadership have responded to Expect With Me.

1. Tell me about how patients react to group prenatal care.

- Tell me an example(s) where participation in Expect With Me had a positive impact on a patient’s life?
- Describe any patterns you have observed regarding women who are not as receptive to this kind of care?
- Probe about how each session component (i.e., connect, expect, explore, grow) and specific topics/activities go over with patients - have list handy.

1. Tell me about the roll out of Expect With Me at your site?

- Probe about the who, what, where, when and how information about this model of group prenatal care was shared with staff
- Probe about: trainings that occurred
- What was good and what could be improved about the Expect With Me facilitator training?
  - Was is good and what could be improved about the Facilitator’s guide?
- Probe about how recruitment and scheduling is done and has this changed over time

1. Walk me through a typical session – prep through clean-up/wrap-up –who does what, when, and how

- Probe about: tips/strategies to achieve maximum group participation
- Probe about strategies to streamline time spent on administrative activities before and after group sessions

1. Tell me about your experience with the Expect With Me website?

- Beyond completing session evaluations, how have you used the website?
- Probe about:
  - data on dashboard
  - managing groups
  - managing participants
  - resources
  - discussion prompts
  - entering vitals
- How do you think the website could be made more useful?

1. Tell me about your or [Name of Organization’s] future plans with regard to Expect With Me.

- What do you see as the biggest challenges for your institution to keep offering this model of group prenatal care?
  - What might be done to mitigate or address these challenges?

1. What would you say about Expect With Me, if given an opportunity to speak very candidly, to representatives from other health care institutions considering adopting this model of prenatal care?

- Probe about: what “sales pitch”/”elevator speech” and/or cautionary tale would be.
- What advice would you give other clinics implementing EWM regarding providers who remain skeptical about the value of group prenatal care?
