# Supplementary material for: Group prenatal care successes, challenges, and frameworks for scaling up: a case study in adopting health care innovations
Source: Implement Sci Commun. 2024 Mar 4;5:20. doi: 10.1186/s43058-024-00556-1 (PMC10913654; doi:10.1186/s43058-024-00556-1)
Supplement: Supplementary file 2 — Supplementary Material 2. [file 43058_2024_556_MOESM2_ESM.docx]

**Provider Sub-Study**

**Qualitative Interview / Focus Group Guide**

GROUP PRENATAL CARE

- What do you think about Expect with Me?
  - How has Expect With Me changed your job?
  - What do you see as the biggest advantages to Expect With Me?
  - What do you see as the biggest disadvantages to Expect With Me?
  - Do you think it is beneficial to patients? If not, why not? If so, how so?
  - For whom do you think Expect With Me is appropriate?

TRAINING/READINESS

- What did you like most about the training?
- What would you have changed?
- When you finished the training, did you feel ready to implement Expect With Me?
- What did you feel most confident about with regards to implementing Expect With Me after the training?
- What were your biggest concerns about implementing Expect With Me when you finished the training?
  - Have these concerns been addressed?
- Do you feel like you know what you are doing? If not, what would help you feel more confident?
- What additional training or resources would you like to see available for facilitators?

RECRUITMENT AND SCHEDULING

- How do you feel about scheduling women for group?
  - What could make it easier?
- How do women respond when you tell them about group?
- How does recruitment to group work in your clinic? What do you think of your role in recruitment to Expect With Me? Are women receptive? Is it easy or difficult? What makes it difficult? How do you get around these challenges?

TECHNOLOGY

- What do you think about the website?
- Is it easy or difficult to use? What makes it difficult? How do you get around these challenges?
- What aspects of the website were helpful? What was cumbersome?
- What patient features did you like? Dislike?
- What provider features did you like? Dislike?
- How did you use the resources?
- How did you use the information patients entered?
- What would you change?

IMPLEMENTATION

- What do you think about implementing Expect With Me? Do you enjoy it? Are the women receptive to this model of care? What is the easiest part about implementation?
- Have you had successes or challenges with cultural appropriateness or adaptation in group?
- What difficulties did you experience when implementing Expect With Me?
  - Organizational issues?
  - Logistic (e.g. space or scheduling)
  - Group issues?
- How do you overcome these barriers?
- What do you think about the curriculum?
- What do you think about the self-care activities?
- What do you think about the web interface?
- What did you like most about the materials? What would you change?
- What advantages do you see to adopting this model permanently?
- Do you see challenges do you see to adopting this model permanently? If so, what are they? If not, what do you think makes this model a good fit?
- Demonstrate for me your pitch to join Expect With Me as if I were an interested patient.
- What are the best practices you would recommend for facilitating groups?

INSTITUTIONAL SUPPORT/SUSTAINABILITY

- How do you think Expect With Me fits in at the organizational and policy levels at your clinic?
- Do you see any advantages at your clinic that help Expect With Me work well?
- Do you see any issues at the organizational and policy levels at your clinic?
  - What are they?
- How have the (other) providers responded to Expect With Me? Are they supportive?
- How has the support staff responded to Expect With Me? Are they supportive?
- How has the clinic leadership responded to Expect With Me? Are they supportive?
- How do you think Expect With Me could be made more successful at your site? What changes would you like to see in Expect With Me at your site?

IMPACT

- How does *Expect With Me* influence the interpersonal relationships of pregnant women and their partners, children, other family members, broader social network?
- To what extent do you think *Expect With Me* changes peer and community norms for reducing health risks and increasing health promoting behaviors?
- How do patients respond to HIV/STD messages?
- Have you noticed any impact of the Expect With Me education on:
  - Individual risk and preventive behaviors (sexual & drug risk)?
  - Maintaining a healthy pregnancy?
  - Readiness for labor & delivery?
  - Perinatal and postpartum outcomes?
- Has your experience in Expect With Me changed how you interact with patients in individual care? If so, explain.
- How has Expect With Me changed patient flow within your clinic?

BILLING/ADMINISTRATION

- How was billing handled?
- How was record keeping handled?
- How was setup/breakdown and other administrative duties different in this model than individual care? Did this pose challenges? How did you handle these challenges? How do you think this could be made more successful?

ADDITIONAL QUESTIONS

- What are the key issues that influence the risk of the populations your practice works with?
- Describe your involvement with Expect With Me.
- How do you think Expect With Me has benefited:
  - Your clinic
  - Your staff
  - Your patients
- How was it decided how many providers would participate in Expect With Me?
- Were there any policy level barriers to adopting the Expect With Me program?
  - How did you address them?
- What challenges have you observed with the Expect With Me?
  - What efforts have you made to address them?
- Do you envision expanding the Expect With Me?
  - If yes:
    - When and how do you envision expanding the program?
    - What on-going financial resources do you have to support the program (have you made Expect With Me a line item in your budget)?
    - Do you have a goal for the percent or number of women you would like to see getting care through the Expect With Me?
  - If no:
    - Why not?
- To what extent do you think *Expect With Me* changes peer and community norms for reducing health risks and increasing health-promoting behaviors?
- How do you think Expect With Me could be made more successful?
- Would you recommend Expect With Me to other providers?
- How would you pitch Expect With Me to a new clinic?
- Would you be willing to share your experiences with providers and leadership at other clinics?
- What else would you like to share about your EWM experience?
